# Isolation and Insecticidal Activity of Essential Oil from *Artemisia lavandulaefolia* DC. against *Plutella xylostella*

**DOI:** 10.3390/toxins13120842

**Published:** 2021-11-25

**Authors:** Xing Huang, Yulin Huang, Chunyue Yang, Tiantian Liu, Xing Liu, Haibin Yuan

**Affiliations:** College of Plant Protection, Jilin Agricultural University, Changchun 130118, China; huangxing@jlau.edu.cn (X.H.); huangyulin0208@163.com (Y.H.); y319915876@163.com (C.Y.); ltt15944890963@163.com (T.L.); LX1065610622@163.com (X.L.)

**Keywords:** *Artemisia lavandulaefolia* DC, *Plutella xylostella*, fractionation, insecticidal activity

## Abstract

Many plants show significant biological activity against pests due to their unique chemical constituents. It is important to identify effective constituents for their development and utilization as botanical pesticides. Our previous study showed that *Artemisia lavandulaefolia* essential oil had biological activity against *Plutella xylostella*. Here, we isolated and identified the constituents of essential oil from *A*. *lavandulaefolia* by silica gel column chromatography. The main constituents identified were eucalyptol and caryophyllene oxide, and they were confirmed by gas chromatography–mass spectrometry (GC–MS). Eucalyptol and caryophyllene oxide showed strong contact toxicity against *P. xylostella* larvae after 24 h of application (Median lethal dose, LD_50_ = 76.97 μL/mL and 20.71 mg/mL. Furthermore, the two active constituents against *P. xylostella* adults showed significant fumigant activity (Mmedian lethal concentration, LC_50_ = 3.25 μL/L and 1.06 mg/L, respectively. Finally, we measured the detoxification enzymes and acetylcholinesterase of the larvae treated with active constituents. The eucalyptol-treated larvae displayed enhanced carboxylesterase (CarE) and glutathione-S-transferase (GST) activities in an in vivo experiment, but it was lower for acetylcholinesterase (AchE) activity. The activities of the CarE and GST significantly decreased when exposed to caryophyllene oxide. In general, the two active constituents, eucalyptol and caryophyllene oxide, showed high insecticidal activity, which demonstrates their potential to be used as natural insecticides.

## 1. Introduction

The diamondback moth *Plutella xylostella* Linnaeus (Lepidoptera: Plutellidae) is the most serious pest of cruciferous crops globally and causes economic losses amounting to US$ 4–5 billion annually [1]. China, for instance, spends approximately US$ 0.77 billion for its management [2]. However, the long-term use of chemicals to control *P. xylostella* has resulted in numerous ecological problems, including the decreased effectiveness of these chemical insecticides, residues, and their accumulation as harmful substances [3,4]. Many plant-derived chemicals are used to control pests but have little or no impact on non-target organisms and the environment [5]. One such source is plant essential oils, which are extracted from the roots, stems, leaves, branches, fruits, seeds, flowers, and even the whole plant. Plant essential oils are made up of 20–60 constituents of different concentrations [6,7]. China’s vast territory and unique natural conditions harbor many plant resources from which essential oils have been extracted (more than 400 plant species found in more than 110 genera) [8,9,10].

*Artemisia lavandulaefolia* DC. (Asteraceae) is a common perennial herb that has a strong aroma. It is distributed in many provinces in China. This plant has clinical application value for the treatment of bronchitis, fever, and cholecystitis [11]. In ancient China, it was placed under blankets to repel mosquitoes and insects. Crude extracts of *A. lavandulaefolia* obtained with different solvents showed antifeedant effects on *Lymantria dispar*, and the antifeedant rates reached 89.33% in petroleum ether solvent [12]. The essential oil from *A. lavandulaefolia* was shown to have significant insecticidal effects on *Sitophilus zeamais* (Motsch.) [13]. Moreover, the ethanol extract of *A. lavandulaefolia* was shown to have repellent and antifeedant effects on *Panonychus citri* adults [14]. In our previous research, the bioactivity of the essential oil from *A. lavandulaefolia* on *P. xylostella* was evaluated; we found that it had contact and fumigant activity on *P. xylostella* (LD_50_ = 0.045 μL/larva, LC_50_ = 0.113 mg/L) [15]. However, there are no reports on the specific constituents that have insecticidal functions. Variations occur in the chemical composition of essential oil due to geographic and seasonal factors and the extraction method. Likewise, the main constituents of *A. lavandulaefolia* essential oil differ under different conditions [13], and their identifications have been determined by GC/MS/DS. For example, different constituents, which include caryophyllene, cis-chrysanthenol, β-thujone, cineole, camphor, eucalyptol, borneol, α-terpineol, β-caryophyllene, and β-farnesene have been identified. However, there is no information on the biological activity of compounds from *A. lavandulaefolia* essential oil against *P. xylostella* after separation. Therefore, we studied the biological activity of different fractions from *A. lavandulaefolia* essential oil against *P. xylostella*.

In view of the foregoing, this study determined the active constituents of *A. lavandulaefolia* essential oil, eucalyptol, and caryophyllene oxide, which had shown biological activities against *P. xylostella*. Furthermore, our work also aimed to evaluate the impact of active essential oil compounds on the detoxification enzymes and acetylcholinesterase of the diamondback moth.

## 2. Results

### 2.1. Bioassays of Five Constituents after Separation

The results showed that the neutral constituent was the most active against *P. xylostella* (Table 1). The corrected mortality reached 100% after 8 h of treatment. The contact effects of the alkaline constituent, weakly acidic constituent, and strongly acidic constituent were not obvious, and the aldehyde–ketone constituent did not reveal a contact effect.

The data (Table 2) revealed that the neutral constituent had a strong fumigation effect on adult *P. xylostella* and showed 100% mortality after 2 h of treatment. The fumigation effects of the aldehyde–ketone constituent, strongly acidic constituent, weakly acidic constituent, and alkaline constituent were not obvious, with corrected mortality rates of 21.11%, 20.00%, 13.33%, and 7.22%, respectively.

### 2.2. Bioassay of the Eight Fractions after Separation

Fractions Za through Zh were separated from the neutral constituent. Among them, fraction Zb was the most active compound against *P. xylostella* larvae in the contact toxicity bioassay, with a corrected mortality rate of 84.44% 24 h after application, followed by fractions Za and Zf. The contact effects of fractions Zc, Zd, Ze, Zg, and Zh were weak (Table 3).

Fraction Zh was significantly bioactive as a fumigant (Table 4), and the corrected mortality rate reached 100% after 2 h of treatment. Fraction Zb also produced great fumigation results against adult *P. xylostella* and showed 97.78% mortality after 24 h of treatment. Fractions Za, Zd, Ze, and Zf had very weak toxicity.

### 2.3. Bioassay of Subfraction Zb2 after Separation

In the contact toxicity study, subfraction Zb2 was the most active compound against *P. xylostella* larvae, with a corrected mortality rate of 76.67% 24 h after treatment. Subfractions Zb1, Zb3, and Zb4 also showed a level of contact effect, but the effect was not significant (Table 5).

In the fumigation toxicity study, subfraction Zb2 showed a significant fumigant effect, with a corrected mortality rate of 82.22% after 24 h of treatment. The fumigant toxicities of subfractions Zb1, Zb3, and Zb4 were very weak (Table 6).

### 2.4. Subfraction Composition

**Figure 1:**
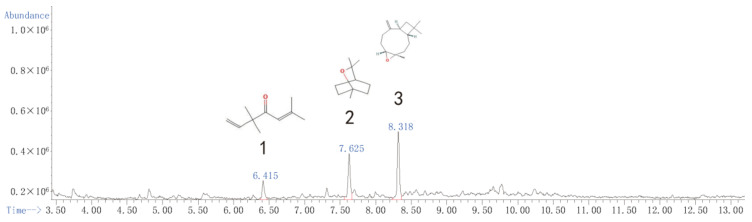
Total ion current chromatogram of the subfraction Zb2 distillate of the essential oil from *A. Lavandulaefolia*.

Although fraction Zh expressed significantly fumigant activity, the contact effects of fraction Zh was weak. We have already added the constituents of Zh in Supplemental Materials (Figure S1 and Table S1). The subfraction Zb2 had great contact and fumigation activity, so we selected fraction Zb to be performed the next separation assey. After the GC–MS analysis, eucalyptol, caryophyllene oxide, and 3,3,6-trimethyl-1,5-heptadiene-4-one were identified as its main constituents (Figure 1 and Table 7). However, although the relative content of 3,3,6-trimethyl-1,5-heptadiene-4-one in subfractions Zb1, Zb3, and Zb4 was very high, they showed low or no insecticidal activity (Figures S2–S4 and Tables S2–S4). Therefore, the biological activities of eucalyptol and caryophyllene oxide were tested in the next experiment.

### 2.5. Bioassay of Eucalyptol and Caryophyllene Oxide

The LD_50_ values of the eucalyptol and caryophyllene oxide toward the *P. xylostella* in the contact toxicity bioassay were 76.97 μL/mL and 20.71 mg/mL, respectively. In the fumigant toxicity bioassay, the LC_50_ values of the eucalyptol and caryophyllene oxide were 3.25 μL/L and 1.06 mg/L, respectively (Table 8).

### 2.6. Effects on Enzymes

Larvae treated with eucalyptol enhanced the CarE and GST activity compared to the control, and there were significant differences among them. However, the activity of AchE was lower and showed no significant effect aside from the treatment with a concentration of 250 μL/mL (Table 9).

The data in the table are mean ± SE (standard error) of three replicates. Significant differences (Tukey’s HSD test *p* ≤ 0.05). The values with different superscripts (^a–^^c^) in the same line are significantly.The activities of CarE and GST significantly decreased in larvae exposed to caryophyllene oxide in comparison to the control. There was no effect on AchE between different concentrations and the control (Table 10).

## 3. Discussion

The insecticidal activities of essential oils have been extensively evaluated, and they have been considered as promising insect control agents. For example, the essential oil from *Artemisia vestita* was shown to possess strong fumigant toxicity and contact toxicity against *Sitophilus zeamais* adults (LC_50_ = 13.42 mg/L air and LD_50_ = 50.62 mg/adult) [16]. Moreover, Sertkaya [17] found that the essential oils of thyme and oregano had a marked acaricidal activity against carmine mite adults (LC_50_ = 0.53 and 0.69 μg/mL air, respectively). Further, the essential oil of *Tephrosia vogelii* was also shown to have a toxic effect against *Cerosipha forbesi,* with an LC_50_ recorded as 0.106 mL/L. Under semi-field conditions, the LC_50_ was estimated to be 0.114 mL/L. Mortality over 90% was observed for all the tested concentrations 24 h after application [18]. Our previous results estimated that the LD_50_ contact toxicity of the essential oil to immature *P. xylostella* was 0.045 μL per larva. *A. Lavandulaefolia* essential oil exhibited fumigant toxicity against *P. xylostella* adults with an LC_50_ of 0.113 mg/L 12 h after treatment [15]. In this study, the neutral constituent from *A. lavandulaefolia* essential oil was the most active against *P. xylostella*. The corrected mortality reached 100% at 250 μL/mL in the contact toxicity bioassays after 8 h of treatment, and the fumigation effect on the adult *P. xylostella* showed 100% mortality at a dose of 50 μL/L within 2 h of exposure.

Although the systematic separation of the essential oil of *A. Lavandulaefolia* should be the first step of determination, many known studies have determined these constituents by GC–MS. For example, eucalyptol [19], α,α,4-trimethyl-3-cyclohexene-1-methanol [13], and borneol [20] were identified as the main constituents of *A. lavandulaefolia* essential oil. Moreover, different collection seasons and geographical environments may result in a variation in the constituents [21]. For instance, caryophyllene (15.5%) and β-thujone (13.8%) were identified as the main constituents of *A. Lavandulaefolia* in central China [22], while caryophyllene (16.1%), cis-chrysanthenol (7.0%), and eucalyptol (5.6%) were identified as the major constituents in Korea. Furthermore, Cha et al. [23] assessed the antimicrobial activity of borneol and eucalyptol on obligate anaerobic bacteria. In our study, the isolation, biological activity screening, chemical separation, and silica gel column chromatography of the constituents from *A. lavandulaefolia* showed that eucalyptol and caryophyllene oxide were the most active compounds.

Many studies have been conducted to assess the insecticidal activities of plant chemical constituents. For example, the chemical composition, insecticidal, and repellent activities of *Artemisia mongolica* essential oil were evaluated against *Lasioderma serricorne* [24]. Among them, 4-Terpineol exhibited the strongest contact activity against *L. serricorne*, showing an LD_50_ value of 8.62 μg/adult. Moreover, camphor and α-terpineol showed stronger fumigant activity (LC_50_ = 2.91 and 3.27 mg/L air, respectively) than crude essential oil and other constituents. At a tested concentration of 39.32 nL/cm^2^, 4-terpineol showed strong repellency (88%), whereas, at 0.06 nL/cm^2^, it attracted insects. In some cases, the dosage was important for whether they could be used as attractants or repellents. Otherwise, the insecticidal activities of Perilla *frutescens* were evaluated against *P. xylostella*. The active ingredient in *P. frutescens* was identified by spectroscopic analysis as the sesquiterpenoid α-farnesene, which showed insecticidal activity against the third-instar larva of *P. xylostella* in a leaf dipping bioassay based on 24-h LD_50_ values (LD_50_ = 53.7 ppm). These results suggested that α-farnesene can be used as a potential control agent against *P. xylostella* [25]. Moreover, the essential oil from *Litsea cubeba* fruits was shown to have strong contact toxicity against *Lasioderma* *serricorne* adults and *Liposcelis bostrychophila*. The main constituents identified by the GC–MS were citral, D-limonene, β-pinene, α-pinene, and linalool. Citral and linalool showed strong contact toxicity (LD_50_ = 11.76, 12.74 μg/adult and 20.15, 99.97 μg/cm^2^, respectively) and fumigant toxicity against *L. serricorne* and *L. bostrychophila* (16.54, 18.04 mg/L air and 0.14, 0.71 mg/L air, respectively) [26]. Our results also showed that eucalyptol and caryophyllene oxide expressed strong contact toxicity against the third-instar larvae of *P. xylostella* (LD_50_ = 76.97 μL/mL and 20.71 mg/mL, respectively) and fumigant activity against *P. xylostella* adults (LC_50_ = 3.25 μL/L and 1.06 mg/L, respectively).

Essential oils are plant-derived volatile substances consisting of a mixture of many compounds. The chief constituents are isoprenoids, along with some aromatic phenols, oxides, ethers, alcohols, esters, aldehydes, and ketones, which are responsible for the smell from essential oil-bearing plants [27,28]. In our study, the active compound, Eucalyptol, also known as 1,8-cineole, is the main constituent of the eucalyptus (*Eucalyptus globulus* Labill.) essential oil and is also found in other plants, such as cardamom (*Elettaria cardamomum* [L.] Maton) and rosemary (*Rosmarinus officinalis* L.) [29]. Eucalyptol is a common monoterpene with the strongest odor. It has medicinal value and can be used to treat bronchial asthma and tumors [30]. It has also been shown to have effects on insects. Our results also showed that eucalyptol performed significant bioactivity against *P. xylostella*, which was in accordance with previous studies. For instance, it was found to be effective against two common storage pests, *Sitophilus oryzae* and *Tribolium castaneum* (Herbst), by fumigation test. Moreover, it showed antifeedant activity against *T. castaneum* [31] and feeding-deterrent effects on *Cydia pomonella* larvae [32]. Further, the antifeedant effects of eucalyptol on *Spodoptera littoralis*, *Myzus persicae*, and *Rhopalosiphum padi* adults were studied in choice tests [33]. Moreover, eucalyptol was identified as one of the main constituents in an extract that showed insecticidal activity [34]. Caryophyllene oxide, an oxygenated sesquiterpene, is present in the families Lamiaceae, Asteraceae, Lauraceae, and Myrtaceae [35]. Extracts for which caryophyllene oxide is the main constituent have been shown to exhibit insecticidal effects. In a direct contact assay, the methanolic extracts of *Syzygium aromaticum* exhibited 67.5% mortality against *P. xylostella* at a 1% (v/v) concentration after 48 h of application [36]. The solvent extracts of *Lantana camara,* which contained caryophyllene oxide, one of its main constituents, showed stronger ovicidal and oviposition deterrent effects against the diamondback moth [37]. The contact and repellent activity of *Cyperus rotundus* essential oil on three stored product pests were also evaluated [38]. Likewise, the repellent activities of essential oils from *Zanthoxylum dimorphophyllum* and *Z. dissitum* obtained by hydrodistillation were evaluated on *Tribolium castaneum* and *Lasioderma serricorne* adults [39]. Generally, the herbivorous insects treated by natural and synthetic insecticides can use detoxification enzymes to metabolize xenobiotics. Detoxification enzymes, including CarE and GST, are generally involved in the metabolism of deleterious secondary plant metabolites in botanical insecticides [40]. AchE catalyzes the breakdown of acetylcholine, which is an important neurotransmitter responsible for the transmission of nerve impulses. In addition to the detoxification enzymes, AchE can also participate in the development of diamondback moth larvae cells by accelerating the development and regeneration of neurons [41,42]. In our study, larvae treated with eucalyptol exhibited excitement, the body turned and twisted, and finally became stiff. The AchE content decreased, and the larvae showed continuous excitement. On the other hand, the activities of the detoxification enzymes, GST and CarE, increased. It might be that the detoxification enzymes in the insects were activated after the treatment with eucalyptol. These results were similar to those of Piri et al. [43] and Visetson et al. [44]. The activity of the CarE and GST significantly decreased in the larvae treated with caryophyllene oxide. This result is in partial agreement with previous reports [45,46,47,48]. The opposite results recorded may be due to different treatment concentrations. The length of the larva became shorter, and the body shrank 24 h after treatment, indicating that there was no effect on the AchE compared to the control.

## 4. Conclusions

On the whole, the essential oil of *A. lavandulaefolia* was separated gradually in this study. The two active compounds, eucalyptol and caryophyllene oxide, showed insecticidal activities against *P. xylostella* in the contact and fumigant bioassays. Furthermore, these two active constituents affect the detoxification enzymes and acetylcholinesterase in insects. All the results demonstrated that these compounds serve as new candidates for the further development into new insecticidal agents. In addition, further research is needed to evaluate the mechanisms underlying their insecticidal activities in order to develop environmentally safe, inexpensive, and agronomically viable insecticides from plant essential oils.

## 5. Materials and Methods

### 5.1. Plant Material Preparation and Essential Oil Extraction

Fresh aerial parts of *A. lavandulaefolia* were collected in September 2019 in Changchun, China (43.8170° N, 125.3235° E). The identity was confirmed by Dr. Tong-Bao Qu, College of Horticulture, Jilin Agricultural University. Voucher specimen (JLH 2158) was deposited at the Herbarium of the College of Horticulture, Jilin Agricultural University. The plants were crushed and then dried in the shade at room temperature for one month. Each 1.5 kg of shade-dried plant material was packed and soaked for 12 h. The crushed aerial parts were subjected to hydrodistillation for 3 h using a Clevenger-type apparatus [49]. The essential oil was placed in a brown bottle and kept sealed at 4 °C.

### 5.2. Test Insects

*Plutella xylostella* were collected from the experimental field of Jilin Agricultural University, Changchun, China. They were reared in a screen cage (43 cm × 43 cm × 43 cm) on *Brassica oleracea* var. *capitata,* which had never been exposed to pesticides. The rearing temperature was 25 ± 1 °C, and 65 ± 5% humidity with the photoperiod at 16:8 (L:D).

### 5.3. Chemicals and Reagents

Exogenou eucalyptol (liquid) and caryophyllene oxide (solid) were evaluated in this study to confirm their biological activities against *P. xylostella*. They were purchased (95–99% purity) from Aladdin (Shanghai, China). Reagents used in the study (NaOH, NaHCO_3_, HCl, and acetone) were obtained from Beijing Chemical Industry, Beijing, China. Silica gel and thin-layer chromatography (TLC) were purchased from Qingdao Marine Chemical Plant, Qingdao, China. Phosphate buffer was purchased from Solarbio, Beijing, China.

### 5.4. Isolation of Essential Oil

The essential oil of *A. lavandulaefolia* was separated preliminarily by chemical methods following the procedures described by Zhongwu Sun [50]. 10 mL essential oil was dissolved in ether and then extracted with different concentrations of chemicals solution (5% NaOH, 10% NaOH, 5% NaHCO_3_, 2% HCl, 5% HCl, and 10% HCl) three times. We finally obtained five constituents, which consisted of an alkaline, strong-acidic, weak-acidic, aldehyde–ketone, and neutral constituent and confirmed which had active effects by bioassays.

The neutral constituent was separated through silica gel column chromatography (200 to 300 mesh, 50 mm inside diameter, 350 mm in length) and eluted using petroleum ether-ethyl acetate (10:0, 15:1, 12:2, 10:3, 10:5, 1:1, 1:2, vol/vol). The separated fractions were collected and concentrated by rotary evaporation (34 °C, 0.05 Mpa). Similar patterns detected by TLC were combined to yield eight fractions (Za through Zh).

The insecticidal activity of the eight fractions was assayed by applying them as fumigants or through contact toxicity bioassays against *P. xylostella*. Although fraction Zh had significant fumigation activity, the contact activity of this ingredient was very weak, so we selected fracton Zb, which has good fumigation and contact effects, for the next test. Fraction Zb was reprocessed by silica gel column chromatography (20 mm inside diameter, 400 mm in length) using petroleum ether–ethyl acetate (10:0, 15:1, 2:1, 1:1, vol/vol) to produce four subfractions (Zb1 through Zb4). The insecticidal activity of Zb1–Zb4 fractions were assessed by bioassays.

### 5.5. Identification of the Active Constituents

The constituents of the *A. lavandulaefolia* essential oil were confirmed by gas chromatography using a GC system (Agilent 6890N, Agilent Technologies Incorporated, Santa Clara, CA, USA), which was equipped with an HP-1 capillary column (30 m × 0.25 mm × 0.25 μm film thickness). The oven temperature was programmed at 60 °C for 3 min, with an increase of 10 °C/min until 280 °C for 5 min. The carrier gas was helium at a flow rate of 1.0 mL/min, the split ratio was 50:1, and the injection volume was 1.0 μL.

The mass spectrometer (Agilent 5975N, Agilent Technologies Incorporated, Santa Clara, CA, USA) used an electron ionization source with 70 eV ionization energy. The ion source temperature was 230 °C, with a scanning range between 20 and 650 *m/z.* The temperature of the quadrupole was 150 °C, and the mass spectrum acquisition delay time was 2 min. The constituents were identified based on their retention index and by the use of the mass spectral libraries (National Institute of Standards and Technology, Nist-2008). The area normalization method was used to calculate the relative content of each constituent.

### 5.6. Bioassays

The biological activity of the constituents on *P. xylostella* was evaluated by applying them as fumigants and through contact toxicity bioassays. The fractions, subfractions, and chemicals from the essential oil were diluted in acetone at a dose of 250 μL/mL, and 1 μL of each preparation was dropped on the dorsal thoracic region of third-instar larvae in independent assays. Treatment with acetone served as the control. Ten larvae were used for treatment under each concentration, which was repeated three times. Control and treatment groups were placed separately in Petri dishes (90 mm diam). All individuals topically treated were maintained in incubators (29 ± 1 °C, 75 ± 5% RH, relative humidity) for observation at 2, 4, 6, 8, 10, 12, and 24 h. The larvae were considered dead if they were unresponsive when touched with a brush. Mortality was calculated as follows:

MR (%) = ND/NA × 100(1)

CM (%) = (MRT − MRC)/(1 − MRC) × 100(2)

where MR: mortality rate, ND: number of dead insects, NA: number of insects treated, CM: corrected mortality, MRT: the mortality rate of the treatments, and MRC: the mortality rate of control.

Fumigant toxicity was assessed on three-day-old *P. xylostella* adults. The constituents, fractions, and subfractions were diluted in acetone at a dose of 50 μL/L. Application of acetone served as the control. Ten μL of each sample was placed on a filter paper (10 cm × 1.5 cm). After the solvent evaporated, the filter paper was placed in a 60 mL glass bottle with 10 adults and the bottle closed to form a sealed chamber. All experiments were performed in incubators (29 ± 1 °C, 75 ± 5% RH) for observation at 2, 4, 6, 8, 10, 12, and 24 h. All tests were conducted in triplicate. The adults were considered dead if they were unresponsive when the bottles were shaken.

### 5.7. Median Lethal Dose (LD_50_) and Median Lethal Concentration (LC_50_) of Chemicals

For the contact toxicity bioassay, serial dilutions of eucalyptol (50, 100, 150, 200, 250 μL/mL) and caryophyllene oxide (10, 20, 25, 50, 100 mg/mL) were prepared in acetone for evaluation. For the fumigant toxicity bioassay, concentration of 1.67, 3.33, 5.00, 6.67, 8.33 μL/L of eucalyptol and 0.83, 1.00, 1.17, 1.33, 1.67 mg/L of caryophyllene oxide were prepared separately for evaluation. Mortality was determined after 24 h. The LD_50_ and LC_50_ values of chemicals were determined using probit analysis.

### 5.8. Enzyme Assays

The enzyme activities in test insects on which contact toxicity bioassay was carried out were evaluated. Ten third-instar larvae were obtained and placed in a glass homogenate tube under ice bath conditions to which 0.01 mol/L phosphate buffer (pH 7.0) was added. The homogenate was centrifuged at 12,000× *g* for 30 min at 4 ℃ (Centrifuge 5430R, Eppendorf, Hamburg, Germany), and the supernatant was used for assays of detoxification enzymes (CarE and GST) and acetylcholine esterase. Enzyme assay kits (Nanjing Jiancheng Bioengineering Institute, Nanjing, China) were used to measure the activities of enzymes.

Briefly, for CarE activity, the working fluid was pre-heated at 37 °C for 30 min before determination. Then, about 5 μL distilled water and 1000 μL working fluid were mixed together to serve as blank controls. The test plates contained 5 μL sample and 1000 μL working fluid. The absorbance of all samples was determined at 450 nm after rapid mixing. The first test was carried out after 10 s and the second after 190 s. For AchE activity, about 40 μL of the sample solution was added to a 96-well plate in each test, and the absorbance of each well was measured at 412 nm. Each sample, control, standard, and blank measurement was tested in triplicate, and every well was performed in triplicate. For GST activity, about 100 μL of the sample solution was added to a 96-well plate in each test, and the absorbance of each well was determined at 412 nm. Each sample, control, standard, and blank was tested in triplicate, and every well was measured in triplicate [48].

### 5.9. Statistical Analysis

All data analyses were performed using SPSS Statistics 17.0 (SPSS, Chicago, IL, USA). Data were expressed as mean ± standard error of the mean. The differences between the constituents were evaluated using one-way ANOVA. Post hoc analysis was carried out using Tukey’s HSD test at 5% significance. Analysis of 50% mortality (LC_50_ and LD_50_) resulting from the treatment with eucalyptol and caryophyllene oxide was determined by log-probit analysis containing 95% fiducial limits.

## Figures and Tables

**Table 1:** Mean ± SE percentages of larvae *P. xylostella* mortality in contact toxicity bioassays with five constituents of *A. lavandulaefolia* essential oil at different times.

Treatment Time (h)	Neutral Constituent	Alkaline Constituent	Weak Acid Constituent	Strong Acidic Constituent	Aldehyde Ketone Constituent
2	89.44 ± 0.08 ^a^	0.00 ± 0.00 ^b^	0.00 ± 0.00 ^b^	0.00 ± 0.00 ^b^	0.00 ± 0.00 ^b^
4	96.67 ± 0.06 ^a^	0.00 ± 0.00 ^b^	0.00 ± 0.00 ^b^	0.00 ± 0.00 ^b^	0.00 ± 0.00 ^b^
6	99.44 ± 0.02 ^a^	0.00 ± 0.00 ^b^	0.00 ± 0.00 ^b^	0.00 ± 0.00 ^b^	0.00 ± 0.00 ^b^
8	100.00 ± 0.00 ^a^	0.00 ± 0.00 ^b^	0.00 ± 0.00 ^b^	0.00 ± 0.00 ^b^	0.00 ± 0.00 ^b^
10	100.00 ± 0.00 ^a^	0.00 ± 0.00 ^b^	0.00 ± 0.00 ^b^	0.00 ± 0.00 ^b^	0.00 ± 0.00 ^b^
12	100.00 ± 0.00 ^a^	0.00 ± 0.00 ^c^	1.67 ± 0.04 ^b^	0.00 ± 0.00 ^c^	0.00 ± 0.00 ^c^
24	100.00 ± 0.00 ^a^	0.56 ± 0.02 ^c^	3.33 ± 0.05 ^b^	2.22 ± 0.04 ^bc^	0.00 ± 0.00 ^c^

Significant differences (one-way ANOVA, Tukey’s Honestly Significant Difference, HSD test, *p* ≤ 0.05) are indicated by different letters among five constituents at the same treatment time. The values with different superscripts (^a–^^c^) in the same line are significantly.

**Table 2:** Mean ± SE percentages of adult *P. xylostella* mortality in fumigant toxicity bioassays with five constituents of *A. lavandulaefolia* essential oil at different times.

Treatment Time (h)	Neutral Constituent	Alkaline Constituent	Weak Acid Constituent	Strong Acidic Constituent	Aldehyde Ketone Constituent
2	100.00 ± 0.00 ^a^	0.00 ± 0.00 ^b^	0.00 ± 0.00 ^b^	0.00 ± 0.00 ^b^	0.00 ± 0.00 ^b^
4	100.00 ± 0.00 ^a^	0.00 ± 0.00 ^b^	0.00 ± 0.00 ^b^	0.00 ± 0.00 ^b^	0.00 ± 0.00 ^b^
6	100.00 ± 0.00 ^a^	0.00 ± 0.00 ^b^	0.00 ± 0.00 ^b^	0.00 ± 0.00 ^b^	0.56 ± 0.02 ^b^
8	100.00 ± 0.00 ^a^	0.00 ± 0.00 ^b^	0.00 ± 0.00 ^b^	0.56 ± 0.02 ^b^	0.56 ± 0.02 ^b^
10	100.00 ± 0.00 ^a^	0.00 ± 0.00 ^b^	0.00 ± 0.03 ^b^	2.78 ± 0.06 ^b^	1.67 ± 0.04 ^b^
12	100.00 ± 0.00 ^a^	1.67 ± 0.04 ^c^	5.56 ± 0.07 ^bc^	6.11 ± 0.07 ^bc^	7.22 ± 0.08 ^b^
24	100.00 ± 0.00 ^a^	7.22 ± 0.06 ^c^	13.33 ± 0.11 ^bc^	20.00 ± 0.13 ^b^	21.11 ± 0.11 ^b^

Significant differences (one-way ANOVA, Tukey’s HSD test, *p* ≤ 0.05) are indicated by different letters among five constituents at the same treatment time. The values with different superscripts (^a–^^c^) in the same line are significantly.

**Table 3:** Mean ± SE percentages of larvae *P. xylostella* mortality in contact toxicity bioassays with fractions Za–Zh from the neutral constituent at different times.

Treatment Time (h)	Za	Zb	Zc	Zd	Ze	Zf	Zg	Zh
2	28.89 ± 0.06 ^b^	50.00 ± 0.05 ^a^	15.56 ± 0.05 ^c^	14.44 ± 0.09 ^c^	7.78 ± 0.04 ^c^	52.22 ± 0.08 ^a^	11.11 ± 0.03 ^c^	8.89 ± 0.03 ^c^
4	36.67 ± 0.07 ^b^	55.56 ± 0.07 ^a^	21.11 ± 0.03 ^c^	16.67 ± 0.07 ^cd^	7.78 ± 0.04 ^e^	56.67 ± 0.10 ^a^	16.67 ± 0.05 ^cd^	10.00 ± 0.00 ^e^
6	41.11 ± 0.11 ^b^	60.00 ± 0.09 ^a^	23.33 ± 0.05 ^c^	26.67 ± 0.09 ^c^	10.00 ± 0.00 ^d^	60.00 ± 0.13 ^a^	18.89 ± 0.03 ^cd^	11.11 ± 0.03 ^d^
8	50.00 ± 0.12 ^b^	67.78 ± 0.07 ^a^	27.78 ± 0.04 ^c^	27.78 ± 0.08 ^c^	13.33 ± 0.05 ^d^	63.33 ± 0.11 ^a^	23.33 ± 0.05 ^cd^	14.44 ± 0.05 ^d^
10	54.44 ± 0.10 ^b^	72.22 ± 0.07 ^a^	30.00 ± 0.00 ^c^	28.89 ± 0.09 ^cd^	18.89 ± 0.08 ^de^	64.44 ± 0.12 ^a^	26.67 ± 0.05 ^cd^	16.67 ± 0.05 ^e^
12	60.00 ± 0.07 ^c^	78.89 ± 0.06 ^a^	34.44 ± 0.05 ^d^	31.11 ± 0.09 ^de^	24.44 ± 0.05 ^ef^	70.00 ± 0.11 ^b^	32.22 ± 0.04 ^de^	20.00 ± 0.05 ^f^
24	62.22 ± 0.07 ^c^	84.44 ± 0.07 ^a^	37.78 ± 0.06 ^d^	36.67 ± 0.01 ^d^	27.78 ± 0.04 ^de^	74.44 ± 0.09 ^b^	36.67 ± 0.07 ^d^	22.22 ± 0.07 ^e^

Significant differences (one-way ANOVA, Tukey’s HSD test, *p* ≤ 0.05) are indicated by different letters among fractions Za-Zh at the same treatment time. The values with different superscripts (^a–^^f^) in the same line are significantly.

**Table 4:** Mean ± SE percentages of adult *P. xylostella* mortality in fumigant toxicity bioassays with fractions Za–Zh from the neutral constituent at different times.

Treatment Time (h)	Za	Zb	Zc	Zd	Ze	Zf	Zg	Zh
2	0.00 ± 0.00 ^d^	47.78 ± 0.08 ^b^	18.89 ± 0.08 ^c^	0.00 ± 0.00 ^d^	0.00 ± 0.00 ^d^	0.00 ± 0.00 ^d^	22.22 ± 0.04 ^c^	100.00 ± 0.00 ^a^
4	0.00 ± 0.00 ^e^	52.22 ± 0.04 ^b^	18.89 ± 0.08 ^d^	0.00 ± 0.00 ^e^	0.00 ± 0.00e	0.00 ± 0.00 ^e^	23.33 ± 0.05 ^c^	100.00 ± 0.00 ^a^
6	0.00 ± 0.00 ^d^	54.44 ± 0.08 ^b^	28.89 ± 0.11 ^c^	0.00 ± 0.00 ^d^	0.00 ± 0.00 ^d^	0.00 ± 0.00 ^d^	27.78 ± 0.04 ^c^	100.00 ± 0.00 ^a^
8	0.00 ± 0.00 ^d^	64.44 ± 0.07 ^b^	32.22 ± 0.11 ^c^	0.00 ± 0.00 ^d^	0.00 ± 0.00 ^d^	0.00 ± 0.00 ^d^	33.33 ± 0.05 ^c^	100.00 ± 0.00 ^a^
10	0.00 ± 0.00 ^d^	67.78 ± 0.08 ^b^	41.11 ± 0.11 ^c^	1.11 ± 0.03 ^d^	0.00 ± 0.00 ^d^	0.00 ± 0.00 ^d^	36.67 ± 0.07 ^c^	100.00 ± 0.00 ^a^
12	0.00 ± 0.00 ^e^	83.33 ± 0.11 ^b^	51.11 ± 0.15 ^c^	3.33 ± 0.05 ^e^	3.33 ± 0.05 ^e^	0.00 ± 0.00 ^e^	41.11 ± 0.06 ^d^	100.00 ± 0.00 ^a^
24	4.44 ± 0.05 ^c^	97.78 ± 0.04 ^a^	58.89 ± 0.11 ^b^	7.78 ± 0.04 ^c^	5.56 ± 0.05 ^c^	2.22 ± 0.04 ^c^	52.22 ± 0.07 ^b^	100.00 ± 0.00 ^a^

Significant differences (one-way ANOVA, Tukey’s HSD test, *p* ≤ 0.05) are indicated by different letters among fractions Za–Zh at the same treatment time. The values with different superscripts (^a–^^e^) in the same line are significantly.

**Table 5:** Mean ± SE percentages of larvae *P. xylostella* mortality in contact toxicity bioassays with subfractions Zb1–Zb4 from fractions Zb at different times.

Treatment Time (h)	Zb1	Zb2	Zb3	Zb4
2	0.00 ± 0.00 ^d^	28.89 ± 0.06 ^a^	11.11 ± 0.03 ^b^	5.56 ± 0.05 ^c^
4	0.00 ± 0.00 ^c^	34.44 ± 0.09 ^a^	13.33 ± 0.05 ^b^	8.88 ± 0.03 ^b^
6	0.00 ± 0.00 ^d^	42.22 ± 0.08 ^a^	16.67 ± 0.05 ^b^	10.00 ± 0.05 ^c^
8	1.11 ± 0.03 ^d^	50.00 ± 0.07 ^a^	22.22 ± 0.07 ^b^	12.22 ± 0.07 ^c^
10	2.22 ± 0.04 ^d^	58.89 ± 0.08 ^a^	25.56 ± 0.05 ^b^	15.56 ± 0.07 ^c^
12	4.44 ± 0.05 ^d^	67.78 ± 0.10 ^a^	27.78 ± 0.07 ^b^	17.78 ± 0.04 ^c^
24	10.00 ± 0.05 ^d^	76.67 ± 0.09 ^a^	32.22 ± 0.04 ^b^	18.89 ± 0.06 ^c^

Significant differences (one-way ANOVA, Tukey’s HSD test, *p* ≤ 0.05) are indicated by different letters among subfractions Zb1–Zb4 at the same treatment time. The values with different superscripts (^a–^^d^) in the same line are significantly.

**Table 6:** Mean ± SE percentages of adult *P. xylostella* mortality in fumigant toxicity bioassays with subfractions Zb1–Zb4 from fractions Zb at different times.

Treatment Time (h)	Zb1	Zb2	Zb3	Zb4
2	0.00 ± 0.00 ^b^	18.89 ± 0.03 ^a^	0.00 ± 0.00 ^b^	0.00 ± 0.00 ^b^
4	0.00 ± 0.00 ^b^	27.78 ± 0.08 ^a^	0.00 ± 0.00 ^b^	0.00 ± 0.00 ^b^
6	0.00 ± 0.00 ^b^	37.78 ± 0.04 ^a^	3.33 ± 0.05 ^b^	0.00 ± 0.00 ^b^
8	0.00 ± 0.00 ^c^	47.78 ± 0.07 ^a^	7.78 ± 0.04 ^b^	0.00 ± 0.00 ^c^
10	0.00 ± 0.00 ^c^	56.67 ± 0.07 ^a^	11.11 ± 0.03 ^b^	2.22 ± 0.04 ^c^
12	1.11 ± 0.03 ^c^	66.67 ± 0.09 ^a^	15.56 ± 0.05 ^b^	4.44 ± 0.07 ^b^
24	1.11 ± 0.03 ^c^	82.22 ± 0.07 ^a^	18.89 ± 0.06 ^b^	6.67 ± 0.07 ^b^

Significant differences (one-way ANOVA, Tukey’s HSD test, *p* ≤ 0.05) are indicated by different letters among subfractions Zb1–Zb4 at the same treatment time. The values with different superscripts (^a–^^c^) in the same line are significantly.

**Table 7:** Chemical constituents and relative contents of the subfraction Zb2 distillate of the essential oil from *A. lavandulaefolia*.

No.	Retention Time (min)	Name of Constituent	Molcular Formula	RI ^1^	Relative Content (%)	Identification Method
1	6.417	1,5-Heptadien-4-one, 3,3,6-trimethyl	C_10_H_16_O	1148	14.62	RI, MS ^2^
2	7.623	Caryophyllene oxide	C_15_H_24_O	1172	36.78	RI, MS
3	8.318	Eucalyptol	C_10_H_18_O	1680	48.60	RI, MS

^1^ RI: Retention index, as determined on an HP-1 MS capillary column using the homologous series of ***n***-hydrocarbons. ^2^ MS: Mass Spectrometry.

**Table 8:** Median lethal dose (LD_50_) and median lethal concentration (LC_50_) resulting from the treatment with eucalyptol and caryophyllene oxide on *P.* *xylostella* after 24 h application.

Compounds	N ^1^	Slope ± SE ^2^	LD_50_ ^3^ (95% FL ^4^)	λ^2^	Slope ± SE	LC_50_ ^3^ (95% FL^b^)	λ^2^
Eucalyptol	150	4.39 ± 0.46	76.97 (28.74–111.79)	12.37	5.44 ± 0.51	3.25 (2.37–4.05)	6.371
Caryophyllene oxide	150	6.23 ± 0.58	20.71 (16.36–25.48)	5.863	13.923 ± 1.32	1.06 (0.87–1.21)	13.592

^1^ Total number of larvae or adults used to generate the probit regression estimates; ^2^ standard error; ^3^ LC_50_ and LD_50_ were determined by log-probit analysis; ^4^ fiducial limits.

**Table 9:** Effect of eucalyptol on detoxification enzymes and acetylcholine esterase of third instar of *P. xylostella* larvae by contact toxicity bioassay (*U/mg prot*).

Enzymes	CK ^1^	50 μL/mL	100 μL/mL	150 μL/mL	200 μL/mL	250 μL/mL
CarE	0.193 ± 0.001 ^c^	0.619 ± 0.001 ^bc^	0.864 ± 0.001 ^bc^	0.871 ± 0.051 ^bc^	0.958 ± 0.086 ^b^	1.97 ± 0.055 ^a^
GST	69.022 ± 8.873 ^c^	56.853 ± 4.882 ^bc^	70.363 ± 1.09 ^bc^	76.199 ± 3.107 ^b^	78.464 ± 4.78 ^b^	95.853 ± 6.793 ^a^
AchE	0.064 ± 0.022 ^a^	0.061 ± 0.017 ^a^	0.057 ± 0.012 ^ab^	0.056 ± 0.005 ^ab^	0.046 ± 0.021 ^ab^	0.027 ± 0.018 ^b^

^1^ CK: Treatment with acetone served as the control.

**Table 10:** Effect of caryophyllene oxide on detoxification enzymes and acetylcholine esterase of third instar of *P. xylostella* larvae by contact toxicity bioassay (*U/mg prot*).

Enzymes	CK	10 mg/mL	20 mg/mL	25 mg/mL	50 mg/mL	100 mg/mL
CarE	0.076 ± 0.001 ^a^	0.049 ± 0.001 ^b^	0.038 ± 0.013 ^b^	0.033 ± 0.011 ^b^	0.033 ± 0.001 ^b^	0.031 ± 0.001 ^b^
GST	84.095 ± 10.576 ^a^	61.674 ± 7.944 ^b^	48.267 ± 2.487 ^b^	41.278 ± 2.613 ^b^	42.878 ± 3.989 ^b^	52.927 ± 6.865 ^b^
AchE	0.280 ± 0.068 ^a^	0.280 ± 0.064 ^a^	0.181 ± 0.038 ^a^	0.162 ± 0.006 ^a^	0.154 ± 0.049 ^a^	0.137 ± 0.051 ^a^

The data in the table are mean ± SE (standard error) of three replicates. Significant differences (Tukey’s HSD test *p* ≤ 0.05). The values with different superscripts (^a^^,^^b^) in the same line are significantly.

## Data Availability

Data are contained within the article.

## Supplementary Materials

The following are available online at https://www.mdpi.com/article/10.3390/toxins13120842/s1, Figure S1: Total ion current chromatogram of Zh distillate of essential oil from *A. lavandulaefolia*; Figure S2: Total ion current chromatogram of Zb1 distillate of essential oil from *A. lavandulaefolia*; Figure S3: Total ion current chromatogram of Zb3 distillate of essential oil from *A. Lavandulaefolia*; Figure S4: Total ion current chromatogram of the Zb4 distillate of essential oil from *A. Lavandulaefolia*. Table S1: The main chemical constituents and relative contents of Zh distillate of essential oil from *A. lavandulaefolia*; Table S2: The main chemical constituents and relative contents of Zb1 distillate of essential oil from *A. lavandulaefolia*; Table S3: The main chemical constituents and relative contents of Zb3 distillate of essential oil from *A. lavandulaefolia*; Table S4: The main chemical constituents and relative contents of Zb4 distillate of essential oil from *A. lavandulaefolia*.
