# Supplementary material for: Isolation and Insecticidal Activity of Essential Oil from Artemisia lavandulaefolia DC. against Plutella xylostella
Source: Toxins (Basel). 2021 Nov 25;13(12):842. doi: 10.3390/toxins13120842 (PMC8707372; doi:10.3390/toxins13120842)
Supplement: Supplementary file 1 [file toxins-13-00842-s001.zip › toxins-1458983-supplementary.pdf]

toxins

Supplementary Information

Isolation and Insecticidal Activity of Essential Oil from *Artemisia lavandulaefolia* DC. against *Plutella xylostella*

Xing Huang, Yulin Huang, Chunyue Yang, Tiantian Liu, Xing Liu and Haibin Yuan *

College of Plant Protection, Jilin Agricultural University, Changchun 130118, China; huangxing@jlau.edu.cn (X.H.); huangyulin0208@163.com (Y.H.); y319915876@163.com (C.Y.); ltt15944890963@163.com (T.L.); LX1065610622@163.com (X.L.)
* Correspondence: yuanhaibin@jlau.edu.cn; Tel.: +0431-84333719

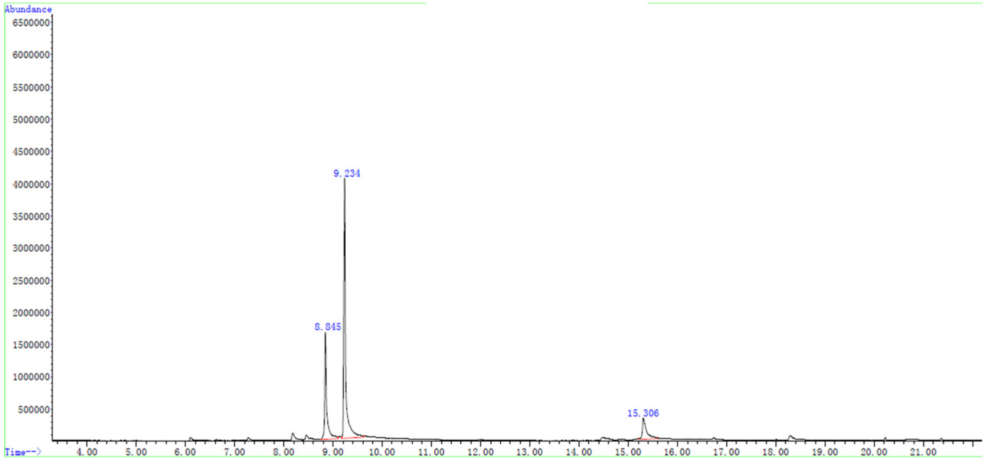

Figure S1. Total ion current chromatogram of Zh distillate of essential oil from *A. lavandulaefolia.*

Table S1. The main chemical constituents and relative contents of Zh distillate of essential oil from *A. lavandulaefolia.*

No.	Retention Time (min)	Name of Constituent	Molcular Formula	Number of CAS	Relative Content (%)
1	6.622	Eucalyptol	$C_{10}H_{18}O$	470-82-6	57.13
2	6.672	Cyclohexanol, 1-methyl-4-(1-methylethenyl)-, acetate	$C_{12}H_{20}O_2$	10198-23-9	32.45
3	8.328	Bicyclo[2.2.1]heptan-2-one, 1,7,7-trimethyl-,(1S)-	$C_{10}H_{16}O$	464-48-2	10.42

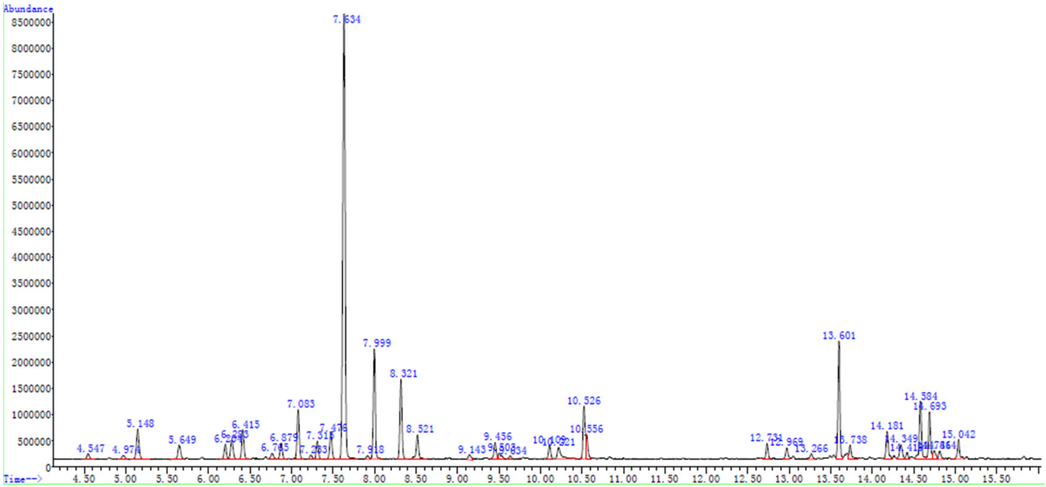

Figure S2. Total ion current chromatogram of Zb1 distillate of essential oil from *A. lavandulaefolia.*

Table S2. The main chemical constituents and relative contents of Zb1 distillate of essential oil from *A. lavandulaefolia.*

No.	Retention Time (min)	Name of Constituent	Molcular Formula	Number of CAS	Relative Content (%)
1	5.150	1R-β-Pinene	$C_{10}H_{16}$	7785-70-8	2.69
2	6.286	β-Pinene	$C_{10}H_{16}$	127-91-3	1.57
3	6.417	β-Myrcene	$C_{10}H_{16}$	123-35-3	2.02
4	7.082	1,3-Cyclohexadiene,1-methyl-4-(1-methylethyl)-	$C_{10}H_{16}$	99-86-5	3.36
5	7.476	β-Phellandrene	$C_{10}H_{16}$	555-10-2	1.88
6	7.630	1,5-Heptadien-4-one,3,3,6-trimethyl-	$C_{10}H_{16}O$	546-49-6	33.68
7	8.001	1,4-Cyclohexadiene,1-methyl-4-(1-methylethyl)-	$C_{10}H_{16}$	99-85-4	6.98
8	8.318	Acetic acid, butyl ester	$C_6H_{12}O_2$	123-86-4	5.14
9	8.519	Cyclohexene,1-methyl-4-(1-methylethylidene)-	$C_{10}H_{16}$	586-62-9	1.76
10	10.528	Bicyclo[2.2.1]heptan-2-one,1,7,7-trimethyl-, (1S)-	$C_{10}H_{16}O$	464-48-2	3.41
11	13.604	3-Hydroxy-3-methylvaleric acid3-	$C_6H_{12}O_3$	150-96-9	7.51
12	14.183	β-Caryophyllene	$C_{15}H_{24}$	6753-98-6	1.99
13	14.585	1,6-Cyclodecadiene,1-methyl-5-methylene-8-(1-methylethyl)-,[s-(E,E)]-	$C_{15}H_{24}$	23986-74-5	4.18
14	14.693	Naphthalene,decahydro-4a-methyl-1-methylene-7-(1-methylethenyl)-,[4aR-(4aπ7π8aπ)]-	$C_{15}H_{24}$	17066-67-0	3.12

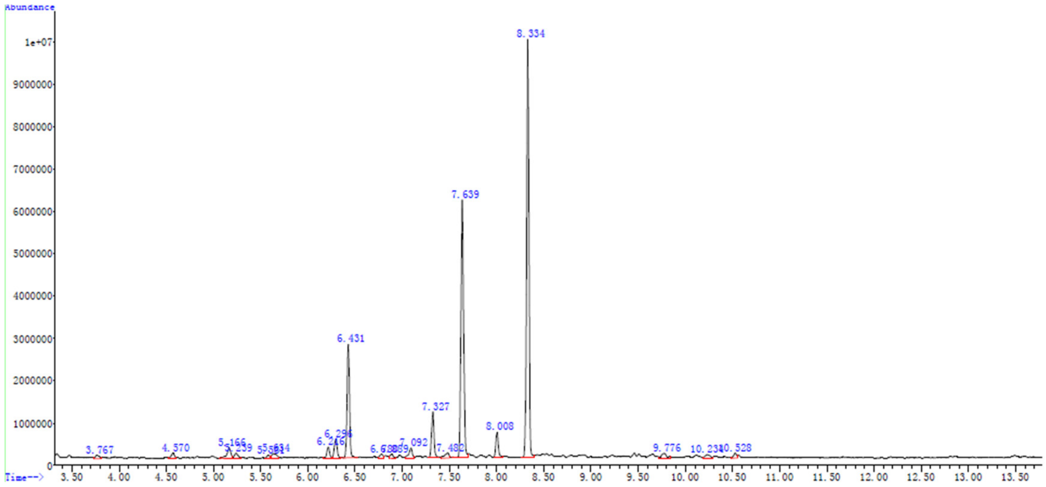

Figure S3. Total ion current chromatogram of Zb3 distillate of essential oil from *A. lavandulaefolia.*

Table S3. The main chemical constituents and relative contents of Zb3 distillate of essential oil from *A. lavandulaefolia.*

No.	Retention Time (min)	Name of Constituent	Molecular Formula	Number of CAS	Relative Content (%)
1	5.634	Eucalyptol	$C_{10}H_{18}O$	470-82-6	0.98
2	6.430	β-Myrcene	$C_{15}H_{24}O_2$	123-35-3	11.02
3	7.326	Limonene	$C_{15}H_{24}O_2$	138-86-3	4.62
4	7.643	Benzene, 1-methyl-4-(1-methylethyl)-	$C_{10}H_{14}$	99-87-6	27.65
5	8.006	1,4-Cyclohexadiene,1-methyl-4-(1-meth-ylethyl)-	$C_{10}H_{16}$	99-85-4	2.67
6	8.338	1,5-Heptadien-4-one, 3,3,6-trimethyl	$C_{10}H_{16}O$	546-49-6	39.23
7	9.775	Caryophyllene oxide	$C_{15}H_{24}O$	1139-30-6	0.94

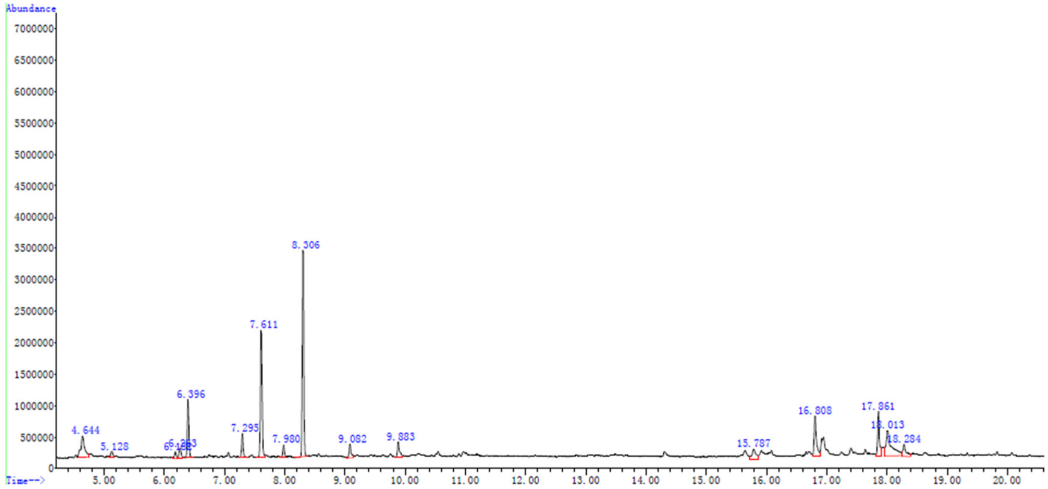

Figure S4. Total ion current chromatogram of the Zb4 distillate of essential oil from *A. lavandulaefolia.*

Table S4. The main chemical constituents and relative contents of Zb4 distillate of essential oil from *A. lavandulaefolia.*

No.	Retention Time (min)	Name of Constituent	Molcular Formula	Number of CAS	Relative Content (%)
1	4.645	1-Pentanol, 4-methyl	$C_6H_{14}O$	626-89-1	6.65
2	6.399	β-Pinene	$C_{10}H_{16}$	127-91-3	7.20
3	7.612	Benzene, 1-methyl-3-(1-methylethyl)-	$C_{10}H_{14}$	535-77-3	17.66
4	8.307	1,5-Heptadien-4-one, 3,3,6-trimethyl	$C_{10}H_{16}O$	546-49-6	24.63
5	16.807	Limonen-6-ol, pivalate	$C_{15}H_{24}O_2$	——	7.34
6	17.858	Cycloundecene, 1-methyl	$C_{12}H_{22}$	88828-82-4	6.11
7	18.013	5-Methoxy-2,2,6-trimethyl-1-(3-methyl-buta-1,3-dienyl)-7-oxa-bicyclo[4.1.0]heptanes	$C_{15}H_{24}O_2$	——	10.91
